# High-Fructose Diet Increases Inflammatory Cytokines and Alters Gut Microbiota Composition in Rats

**DOI:** 10.1155/2020/6672636

**Published:** 2020-11-30

**Authors:** Yong Wang, Wentao Qi, Ge Song, Shaojie Pang, Zhenzhen Peng, Yong Li, Panli Wang

**Affiliations:** ^1^Academy of National Food and Strategic Reserves Administration, Beijing 100037, China; ^2^Department of Nutrition and Food Hygiene, School of Public Health, Peking University, Beijing 100191, China

## Abstract

High-fructose diet induced changes in gut microbiota structure and function, which have been linked to inflammatory response. However, the effect of small or appropriate doses of fructose on gut microbiota and inflammatory cytokines is not fully understood. Hence, the abundance changes of gut microbiota in fructose-treated Sprague-Dawley rats were analyzed by 16S rRNA sequencing. The effects of fructose diet on metabolic disorders were evaluated by blood biochemical parameter test, histological analysis, short-chain fatty acid (SCFA) analysis, ELISA analysis, and Western blot. Rats were intragastrically administered with pure fructose at the dose of 0 (Con), 2.6 (Fru-L), 5.3 (Fru-M), and 10.5 g/kg/day (Fru-H) for 20 weeks. The results showed that there were 36.5% increase of uric acid level in the Fru-H group when compared with the Con group. The serum proinflammatory cytokines (IL-6, TNF-*α*, and MIP-2) were significantly increased (*P* < 0.05), and the anti-inflammatory cytokine IL-10 was significantly decreased (*P* < 0.05) with fructose treatment. A higher fructose intake induced lipid accumulation in the liver and inflammatory cell infiltration in the pancreas and colon and increased the abundances of *Lachnospira*, *Parasutterella*, *Marvinbryantia*, and *Blantia* in colonic contents. Fructose intake increased the expressions of lipid accumulation proteins including perilipin-1, ADRP, and Tip-47 in the colon. Moreover, the higher level intake of fructose impaired intestinal barrier function due to the decrease of the expression of tight junction proteins (ZO-1 and occludin). In summary, there were no negative effects on body weight, fasting blood glucose, gut microbiota, and SCFAs in colonic contents of rats when fructose intake is in small or appropriate doses. High intake of fructose can increase uric acid, proinflammatory cytokines, intestinal permeability, and lipid accumulation in the liver and induce inflammatory response in the pancreas and colon.

## 1. Introduction

Fructose consumption is part of Western diets but is on the rise elsewhere, due to the introduction and generalized use of high-fructose corn syrup (HFCS) in the food industry [1, 2]. A review showed that fructose increased energy intake, reduced insulin sensitivity, increased circulating triglyceride (TG) and visceral fat stores, and depressed energy metabolism compared with glucose or starch [3]. In patients administered with sugary beverages containing high fructose, increasing fat in the liver was detected within the sixth month [4]. Evidence showed that consumption of HFCS in beverages and processed foods induced development of nonalcoholic fatty liver disease (NAFLD) and chronic inflammation [5]. A research suggested that fructose overconsumption-induced inflammation in the visceral adipose tissue *in vivo* could be involved in the development of obesity [6].

Most of the researchers agreed that fructose diet contributed to the risks of obesity, metabolic syndrome, and diabetes mellitus due to excessive consumption [7]. However, Chiu et al. showed that fructose in isocaloric exchange for other carbohydrates had no adverse effects, especially at small or appropriate doses [8]. For example, small doses of fructose (≤50 g/day or ≤10% of total energy intake/day) might improve glycemic control over the long term [9]. Acute clinical evidence demonstrated that small doses (≤10 g/meal) of fructose decreased the postprandial glycemic response in type 2 diabetes patients [10].

The intestinal microbial community has been related to many kinds of metabolic diseases, such as obesity, diabetes, and NAFLD [11–13]. It was reported that high-fructose diet altered the gut microbiota and induced intestinal barrier deterioration *in vivo* [14, 15]. The intestinal metabolite profile is associated with fructose feeding outcomes in reducing bacterial diversity and seems to have more potential in terms of inducing host metabolic disturbances [16]. Results also showed that high-fructose diets induced inflammation and metabolic disorders owing to changes in gut microbiota composition and enhancing intestinal permeability [11]. Mice fed with a 60% fructose diet had alterations of the gut microbiota and intestinal mucosa integrity [17]. However, there is only limited research on the influence of low-dose fructose on gut microbial community and the subsequent effects on inflammatory response and intestinal barrier function.

It appears that the effects of fructose on health are controversial and closely related to intake dose. More physiologically relevant experiments should be designed to generate evidence to better understand the role of fructose on human health. Therefore, different dosages of pure fructose were intragastrically administered to healthy rats over a 20-week period to evaluate the effects of fructose on blood inflammatory cytokines, intestinal barrier function, and gut microbiota in this study.

## 2. Materials and Methods

### 2.1. Materials

Standard substances of acetic, propionic, isobutyric, and butyrate acid were obtained from Sigma-Aldrich (St. Louis, MO, USA). The antibodies perilipin-1, occludin, adipose differentiation-related protein (ADRP), and *β*-actin and secondary antibodies were purchased from Abcam (Cambridge, MA, USA). ZO-1 was obtained from Millipore (Billerica, MA, USA). Tail-interacting protein (Tip-47) was purchased from Santa Cruz (Texas, CA, USA).

### 2.2. Animals

Six-week-old male Sprague-Dawley (SD) rats weighing approximately 220 g were purchased from Beijing Vital River Laboratory Animal Technology (Beijing, China) and acclimatized for 1 week before the experiment. Two rats were in each cage, and they were housed with a 12 h/12 h light/dark cycle at 22°C-25°C and 55%–60% humidity.

### 2.3. Experimental Design

The rats were randomly divided into four groups (*n* = 10): Con group (administration of saline solution), Fru-L group (low dose of fructose, 2.6 g/kg/day), Fru-M group (moderate dose of fructose, 5.3 g/kg/day), and Fru-H group (high dose of fructose, 10.5 g/kg/day). The rats were treated with fructose for 20 weeks until sacrifice. Rats were kept under SPF conditions and were fed with a standard chow diet (Beijing Keao Xieli Feed Co., Ltd., Beijing, China). The energy supply (%) in maintenance diet is as follows: protein 23.07%, fat 11.85%, and carbohydrate 65.08%.

The WHO proposed to halve free sugar intake to 5% of daily calories, which is about 0.83 g/kg/day [18]. Therefore, rats in the Fru-M group were treated with a dose of 5.3 g/kg/day fructose based on the suggested equivalent dose conversion [19]. And the rats in the Fru-L and Fru-H groups were treated with a dose of 2.6 and 10.5 g/kg/day fructose, respectively. A same volume of saline solution was intragastrically administered to the rats in the Con group. Blood glucose levels were measured with a glucometer (LifeScan Inc., Milpitas, CA, USA) every two weeks, and weight were recorded every week. Food consumption was recorded every two days until the end of this study.

### 2.4. Biochemical Parameters

After 20 weeks, the rats were fasted for 12 h and euthanized using CO_2_. The blood samples were collected by cardiac puncture and centrifuged as our previous study [20] and stored at 4°C. The levels of total TG, total cholesterol (TC), low-density lipoprotein cholesterol (LDL-C), high-density lipoprotein cholesterol (HDL-C), aspartate transaminase (AST), alanine aminotransferase (ALT), superoxide dismutase (SOD), malondialdehyde (MDA), lipase, free fatty acid (FFA), and uric acid in serum were determined with the corresponding diagnostic kits (Zhongsheng Beikong Bioengineering Institute, Beijing, China) on a Mindray BS-420 Automatic Analyzer (Shenzhen, China).

### 2.5. Histopathological Examination

Liver, pancreas, and colon tissues were fixed in 10% formalin and then embedded in paraffin. The tissue sections were stained with hematoxylin-eosin and observed by using a BA-9000L microscope (Osaka, Japan). All experiments and scores were performed in a blinded manner by a pathologist in Peking University [21].

### 2.6. Western Blotting

The total protein concentration of colonic mucosal samples was determined using the BCA protein assay kit (Tiangen Biotech, Beijing, China). Western blot analysis was performed according to our previous study [22]. In brief, an equal amount of protein from each group was separated by sodium dodecyl sulfate-polyacrylamide gel electrophoresis and transferred to nitrocellulose (NC) membranes (Millipore, Bedford, MA, USA). Thereafter, the NC membranes were blocked with 5% nonfat dry milk and then were probed with the corresponding primary and secondary antibodies. Finally, proteins were detected and quantified using the ODYSSEY FC imaging system (Gene Company Limited, NE, USA).

### 2.7. Short-Chain Fatty Acid (SCFA) Analysis

Colonic contents were diluted, mixed, and centrifuged, and then, 25% (*w*/*v*) metaphosphoric acid solution was added to the supernatant to extract SCFA. SCFA analysis was performed according to the method previously described using gas chromatography (Agilent 6890, CA, USA) with a DB-FFAP chromatographic capillary column (30 m × 0.25 mm × 0.5 *μ*m; Agilent) [20].

### 2.8. ELISA Analysis

IL-6, TNF-*α*, MIP-2, and IL-10 levels in serum were measured using ELISA kits (R&D Systems, Minneapolis, MN, USA) and a 2300 EnSpire Multimode Plate Reader (PerkinElmer, Waltham, MA, USA) according to the manufacturer's instructions.

### 2.9. Gut Microbiota Analysis

Colonic contents were collected at week 20 and stored at -80°C until analysis. The microbiomes were analyzed at the Novogene Bioinformatics Technology Co., Ltd. (Beijing, China) on an Illumina MiSeq platform. DNA was amplified by using the 515f/806r primer set (515f: 5′-GTGCCAGCMGCCGCGGTAA-3′, 806r: 5′-XXXXXXGGACTACHVGGGTWTCTAAT-3′), which targets the V4 region of bacterial 16S rRNA. Sequencing libraries were generated and analyzed as previously described [20]. Paired-end reads from the original DNA fragments are merged by using FLASH. Sequences were analyzed using QIIME software package, and in-house Perl scripts were used to analyze alpha (Observed_species and Shannon index) and beta diversity.

### 2.10. Statistical Analysis

Data are expressed as the mean ± standard deviation (SD). Statistical analyses were performed using GraphPad Prism Software 5.0 (La Jolla, CA, USA). Differences were analyzed by one-way analysis of variance (ANOVA) with Dunnett's contrast for multiple group comparisons. *P* value < 0.05 was considered significant.

## 3. Results

### 3.1. Effect of Fructose on Body Weight, Feed Intake, and Fasting Blood Glucose

**Figure 1:**
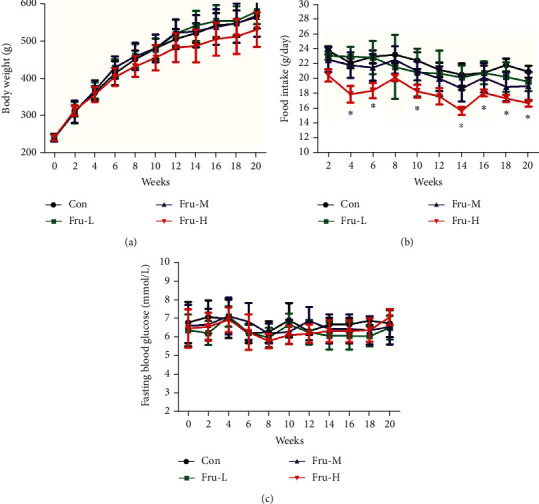
(a) Body weight, (b) food intake, and (c) fasting blood glucose of rats with different doses of fructose during 20 weeks. Con group, administration of saline solution; Fru-L group, administration of a low dosage of fructose (2.6 g/kg/day); Fru-M group, administration of a moderate dosage of fructose (5.3 g/kg/day); Fru-H group, administration of a high dosage of fructose (10.5 g/kg/day). Data are presented as the mean ± SD (*n* = 10). ^∗^*P* < 0.05 means significantly different from the Con group.

Rats treated with fructose in different dosages had similar weight gains to those in the Con group over the 20-week period (Figure 1(a)). The weight gain of rats in the Fru-H group had a lower trend than that in the other groups, although there were no significant differences (*P* > 0.05) during the whole period. It was interesting that the food intake of rats in the Fru-H group was significantly lower than that in the Con, Fru-L, and Fru-M groups in almost the whole period (*P* < 0.05) (Figure 1(b)). There were no significant differences (*P* > 0.05) on fasting blood glucose levels between different groups during 20 weeks (Figure 1(c)).

### 3.2. Effect of Fructose on Serum Biochemical Parameters

TC levels in serum in all the fructose groups were found significantly lower than those in the Con group (*P* < 0.05) (Table 1). The uric acid level in both the Fru-L and Fru-M groups had no significant difference (*P* > 0.05) compared with that in the Con group, while the uric acid level was increased 36.5% in the Fru-H group when compared with the Con group (*P* < 0.05). There were no significant differences on other blood parameters including TG, HDL-C, LDL-C, AST, ALT, SOD, MDA, lipase, and FFA (*P* > 0.05).

### 3.3. Effect of Fructose on Inflammatory Cytokines in Serum

**Figure 2:**
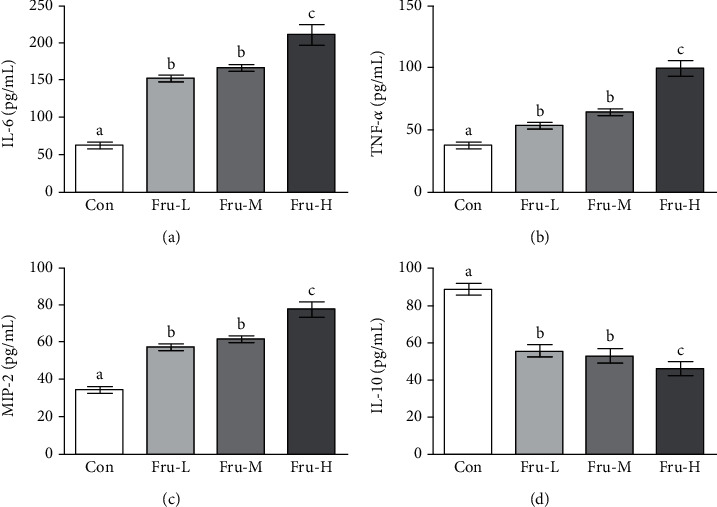
Concentrations of inflammatory cytokines including (a) IL-6, (b) TNF-*α*, (c) MIP-2, and (d) IL-10 in the serum. Con group, administration of saline solution; Fru-L group, administration of a low dosage of fructose (2.6 g/kg/day); Fru-M group, administration of a moderate dosage of fructose (5.3 g/kg/day); Fru-H group, administration of a high dosage of fructose (10.5 g/kg/day). Data are presented as the mean ± SD (*n* = 5). Values that do not share the same lowercase letter are significantly different (*P* < 0.05).

The inflammatory cytokines (IL-6, TNF-*α*, MIP-2, and IL-10) in serum were further determined (Figure 2). The concentrations of IL-6, TNF-*α*, and MIP-2 were all significantly higher in the Fru-L, Fru-M, and Fru-H groups than in the Con group (*P* < 0.05). The levels of IL-6, TNF-*α*, and MIP-2 increased along with fructose dosage, whereas the concentrations of IL-10 were significantly lower in the Fru-L, Fru-M, and Fru-H groups than in the Con group (*P* < 0.05). The levels of IL-10 were decreased as the fructose dosage increases.

### 3.4. Effect of Fructose on Liver and Pancreas Histology

**Figure 3:**
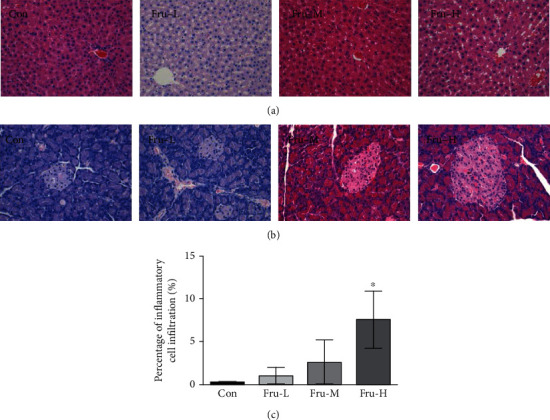
Effect of fructose in different dosages on the liver and pancreas of rats. (a) Representative micrographs of HE-stained sections of the liver (×40); (b) representative micrographs of HE-stained sections of the pancreas (×40); (c) scores based on inflammatory cell infiltration in the pancreas. Con group, administration of saline solution; Fru-L group, administration of a low dosage of fructose (2.6 g/kg/day); Fru-M group, administration of a moderate dosage of fructose (5.3 g/kg/day); Fru-H group, administration of a high dosage of fructose (10.5 g/kg/day). Data are presented as the mean ± SD (*n* = 5). ^∗^*P* < 0.05 means significantly different from the Con group.

In the Fru-H group, 10% hepatic microvesicular steatosis was observed, while no obvious microvesicular steatosis was observed in the other groups (Figure 3(a)). Tissue necrosis and inflammatory cell infiltration could be found in the pancreas of rats in the Fru-M and Fru-H groups, while the pancreatic constructions of rats in the Con and Fru-L groups were very clear and no obvious pathology damage (Figure 3(b)). The score based on the inflammatory cell infiltration in the pancreas of the Fru-H group was significantly higher than that of the Con group (*P* < 0.05), while the Fru-L and Fru-M groups showed no significant difference compared with the Con group (*P* > 0.05, Figure 3(c)).

### 3.5. Effect of Fructose on Colon Histology

**Figure 4:**
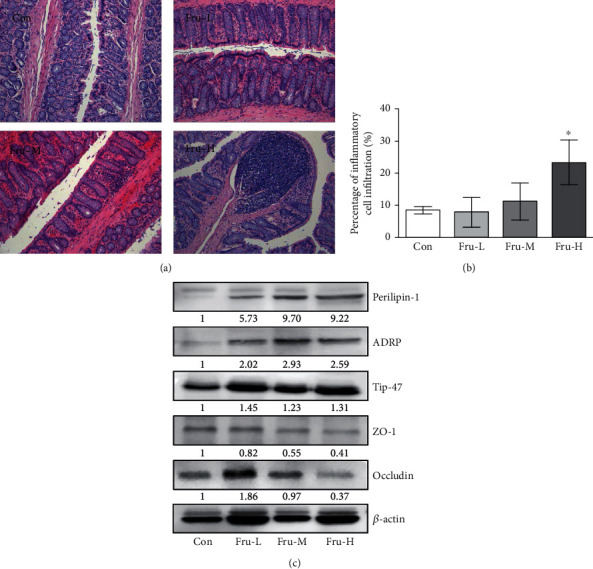
Effect of fructose in different dosages on the colon of rats. (a) Representative micrographs of HE-stained sections of rat colon (×40); (b) scores based on inflammatory cell infiltration in the colon. Data are presented as the mean ± SD (*n* = 5). ^∗^*P* < 0.05 means significantly different from the Con group; (c) Western blot bands of perilipin-1, ADRP, Tip-47, ZO-1, and occludin in the colon. Con group, administration of saline solution; Fru-L group, administration of a low dosage of fructose (2.6 g/kg/day); Fru-M group, administration of a moderate dosage of fructose (5.3 g/kg/day); Fru-H group, administration of a high dosage of fructose (10.5 g/kg/day).

Inflammatory cell infiltration can be observed in the mucoderm and submucosa in the Fru-H group, while no obvious inflammatory cell infiltration was observed in those of other groups (Figure 4(a)). The percentage of inflammatory cell infiltration in the colon of the Fru-H group was significantly higher than that of the Con group (*P* < 0.05, Figure 4(b)), while there were no significant differences among the Con, Fru-L, and Fru-M groups (*P* > 0.05).

### 3.6. Effect of Fructose on the Expressions of Lipid Accumulation and Tight Junction (TJ) Proteins

Proteins related to lipid accumulation and TJ were further examined by Western blot. The results showed that the expressions of lipid accumulation proteins including perilipin-1, ADRP, and Tip-47 were increased obviously, while the expressions of TJ proteins including ZO-1 and occludin were decreased especially in the Fru-H group (Figure 4(c)).

### 3.7. Effect of Fructose on Gut Microbiota and SCFAs in Colonic Contents

**Figure 5:**
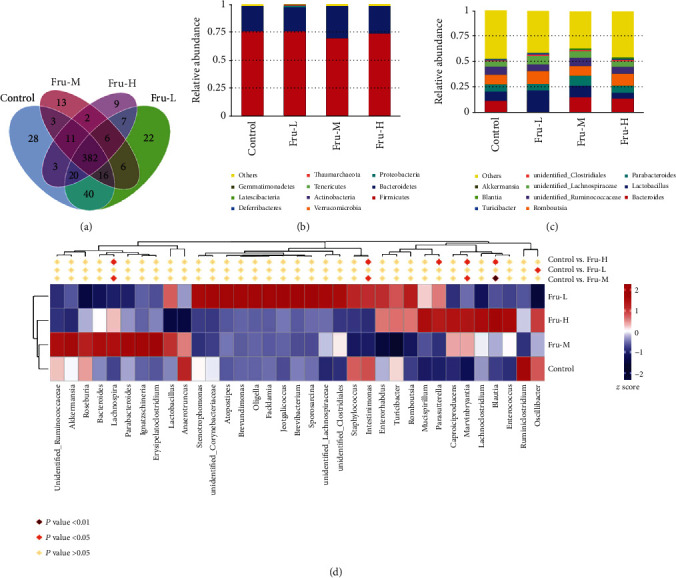
Effect of fructose in different dosages on gut microbiota of rats. (a) Venn diagram displays OTUs among the four groups in colonic contents (*n* = 6 per group); (b) analysis of relative abundance of microbiota at the phylum level in colonic contents; (c) analysis of relative abundance of microbiota at the genus level in colonic contents; (d) heatmaps of genus. Control, administration of saline solution; Fru-L group, administration of a low dosage of fructose (2.6 g/kg/day); Fru-M group, administration of a moderate dosage of fructose (5.3 g/kg/day); Fru-H group, administration of a high dosage of fructose (10.5 g/kg/day).

To compare community structure and similarity of gut microbiota, 16S rRNA analysis was carried out in this research. The shared OTUs for different groups were determined via the Venn diagram (Figure 5(a)). A total of 382 OTUs (67.3%) could be detected in all groups. The Con group had more unique OTUs (28) than the other groups including Fru-L (22), Fru-M (13), and Fru-H (9). The different OTUs between Con and Fru-L, Con and Fru-M, and Con and Fru-H were 86, 118, and 118, respectively. No significant differences were found at the phylum level among these four groups (Figure 5(b)). *Lachnospira*, *Intestinimonas*, *Parasutterella*, *Marvinbryantia*, *Blantia*, and *Oscilibacter* abundances at the genus level were significantly differential under the effect of fructose (Figures 5(c) and 5(d)). *Lachnospira* and *Marvinbryantia* were significantly enriched in the Fru-M and Fru-H groups compared with the Con group (*P* < 0.05). *Parasutterella* and *Blantia* were significantly increased in the Fru-H group compared with the Con group (*P* < 0.05), but *Blantia* was significantly decreased in the Fru-M group compared with the Con group (*P* < 0.05). *Intestinimonas* was significantly decreased in the Fru-M and Fru-H groups compared with the Con group (*P* < 0.05). *Oscilibacter* was significantly decreased in the Fru-L group compared with the Con group (*P* < 0.05).

SCFAs in colonic contents of rats were further examined. There were no significant differences (*P* > 0.05) on acetic, propionic, and butyric acid and total SCFAs in all groups (Table 2). However, the concentration of isobutyric acid was relatively lower in all the fructose-treated groups than the Con group (*P* < 0.05).

## 4. Discussion

The effect of fructose on health has been controversial and closely related to its intake dosage. In this study, rats were intragastrically administered with fructose at the dosages of 2.6, 5.3, and 10.5 g/kg/day, respectively. It was found that the diet consumption of rats significantly decreased in the Fru-H group. As for the reason, we supposed that high fructose intake provided energy to those rats and decreased diet consumption. The body weight and epididymal fat index (data not shown) of rats in the Fru-H group were lower than those in the Con group. This could be the reason why TC levels were decreased with the treatment of fructose. Nevertheless, this related mechanism needs to be investigated in future works. Our data also showed that there were no changes on fasting blood glucose of rats after fructose intake during the whole feeding period.

A previous study showed that long-term fructose consumption increased serum uric acid concentration [23]. In the present study, a 36.5% increase of serum uric acid level was found in high fructose intake rats compared with control rats. Here, we found that there were no significant differences in serum levels of uric acid between control rats and small or mild fructose-fed rats which means the risk of hyperuricemia with appropriate fructose intake is not high. Meta-analysis showed that “catalytic” small fructose doses might improve glycemic control without adverse effects on body weight and uric acid [9, 24].

Many factors such as uric acid generation and high sugar exposure may accelerate the fructose-induced NAFLD [25]. Our results showed that there was no obvious inflammatory response in the liver after intake of fructose. However, mild microvesicular steatosis was detected at the highest dose of fructose, which indicated a higher risk of fatty liver induced by high fructose, even though the function of the liver has not been affected at that time.

It has been demonstrated that fructose-induced metabolic syndrome is closely related to inflammation, characterized by increased proinflammatory cytokine concentration and inflammation signaling activation in the organs (liver, pancreatic islet, and intestine) [26]. Superabundant fructose alters the gut microbiota composition and impairs intestinal barrier function via decreased expression of TJ proteins, therefore initiating inflammatory process [11, 27]. High fructose has been found with the capability to induce inflammation chemokine overproduction, such as IL-1*β*, TNF-*α*, and IL-6 [28]. In the present study, we discovered that fructose significantly increased the levels of plasma proinflammatory markers IL-6, TNF-*α*, and MIP-2 in a dose-dependent manner and decreased the level of anti-inflammatory marker IL-10 in rats. In addition, some organs including the pancreas and colon showed significant inflammatory symptoms at the highest doses of fructose in this study.

In this research, we found significant inflammatory symptoms in the colon of rats with the highest fructose intake. Li et al. revealed high-fructose diet-induced intestinal epithelial barrier dysfunction in mice, and SCFAs could improve intestinal barrier function [27]. SCFAs are the end products of microbial fermentation of nondegradable carbohydrates and can inhibit intestinal inflammation [29, 30]. A high-sugar diet could reduce SCFA production in mice, decrease microbial diversity, and thus enhance susceptibility to colitis [31]. In our study, the isobutyric acid and total SCFAs showed a decreased trend in the fructose intake group.

PAT proteins, such as perilipin, ADRP and Tip-47, could induce cellular lipid stores [32]. In this paper, we found that the expressions of perilipin, ADRP, and Tip-47 were all induced by high levels of fructose intake which means a potential increase of lipid droplets in the colon. We also confirmed that high fructose intake rats exhibited increased gut permeability, characterized by disruption of the TJ proteins (ZO-1 and occludin) in the colon. Increased gut permeability was closely related to obesity and causes inflammation [33]. Therefore, these results indicated that high-level fructose intake induces the extension of the colon, which further leads to increased gut permeability and inflammation.

A previous study has proved that elevated luminal fructose could induce proinflammatory effects via initiating changes in gut microbiota [15]. Higher abundance of proinflammatory taxa *Marvinbryantia* which was associated with intestinal inflammation and bowel dysfunction [34, 35] was observed in the Fru-M and Fru-H groups. Increased abundances of *Lachnospira*, *Parasutterella*, and *Blantia* in colonic contents were detected, while *Lachnospira*, an acetate-producing bacteria, was related to increasing blood glucose levels [36], and *Parasutterella*, known as the saccharolytic strain, was increased by carbohydrate consumption with a potential role on bile acid maintenance and intestinal mucosal homeostasis [37, 38]. *Blantia* had positive anti-inflammatory effects and had the ability to produce SCFAs [39]. While high-fructose diet altered the abundance of certain taxa, both overall alpha diversity (Observed_species and Shannon index) and beta diversity of colonic content samples among the four groups were not significantly different in this study (data not shown).

In summary, we demonstrated a potential connection between fructose-rich diet, gut microbiota profile, and inflammatory process in the liver, pancreas, and colon. There were no negative effects on body weight, fasting blood glucose, histological analysis, gut microbiota, and SCFAs in the colon when fructose intake was in small or appropriate doses. High fructose intake could induce uric acid increase, lipid accumulation in the liver, and an inflammatory response in the pancreas and colon and increase the abundances of *Lachnospira*, *Parasutterella*, *Marvinbryantia*, and *Blantia* in colonic contents. All doses of fructose dysregulated the inflammatory cytokine production in serum and the expressions of lipid accumulation and TJ proteins in the colon. Further studies should be undertaken to explore more details and mechanisms of fructose intake on health effects.

## Figures and Tables

**Table 1:** Blood biochemical parameters of rats at the end of 20 weeks.

	Con	Fru-L	Fru-M	Fru-H
TG (mmol/L)	1.31 ± 0.26^a^	1.56 ± 0.83^a^	1.67 ± 0.71^a^	0.99 ± 0.12^a^
TC (mmol/L)	2.84 ± 0.48^a^	2.36 ± 0.30^b^	2.42 ± 0.41^b^	2.18 ± 0.28^b^
HDL-C (mmol/L)	1.68 ± 0.23^a^	1.49 ± 0.14^a^	1.62 ± 0.21^a^	1.49 ± 0.19^a^
LDL-C (mmol/L)	0.65 ± 0.11^a^	0.52 ± 0.15^a^	0.57 ± 0.20^a^	0.47 ± 0.14^a^
AST (U/L)	110.40 ± 24.80^a^	93.01 ± 18.81^a^	104.64 ± 73.97^a^	110.66 ± 37.71^a^
ALT (U/L)	51.90 ± 9.50^a^	49.57 ± 8.65^a^	60.19 ± 38.72^a^	44.96 ± 12.53^a^
SOD (U/mL)	72.80 ± 12.57^a^	70.13 ± 9.99^a^	77.43 ± 11.05^a^	78.72 ± 9.55^a^
MDA (nmol/mL)	4.39 ± 1.03^a^	4.64 ± 0.99^a^	4.15 ± 0.57^a^	3.61 ± 0.82^a^
Lipase (U/mL)	67.32 ± 10.90^a^	68.50 ± 12.00^a^	63.25 ± 6.92^a^	59.52 ± 10.95^a^
FFA (mmol/L)	0.45 ± 0.07^a^	0.50 ± 0.08^a^	0.50 ± 0.09^a^	0.47 ± 0.04^a^
Uric acid (*μ*mol/L)	242.27 ± 94.63^a^	213.04 ± 131.31^ab^	167.98 ± 64.49^a^	330.81 ± 71.48^b^

Con group, administration of saline solution; Fru-L group, administration of a low dosage of fructose (2.6 g/kg/day); Fru-M group, administration of a moderate dosage of fructose (5.3 g/kg/day); Fru-H group, administration of a high dosage of fructose (10.5 g/kg/day). Data are expressed as the mean ± standard deviation (*n* = 6). Values in the same row that do not share the same lowercase letter are significantly different (*P* < 0.05).

**Table 2:** Effects of fructose on SCFA concentrations in the colon of rats at the end of 20 weeks.

	Con	Fru-L	Fru-M	Fru-H
Acetic acid (*μ*g/g)	147.02 ± 39.60^a^	157.52 ± 36.14^a^	126.70 ± 17.20^a^	145.33 ± 28.62^a^
Propionic acid (*μ*g/g)	34.25 ± 9.27^a^	41.92 ± 8.75^a^	29.38 ± 3.74^a^	39.27 ± 6.03^a^
Butyric acid (*μ*g/g)	22.81 ± 8.63^a^	40.49 ± 9.08^a^	38.70 ± 5.28^a^	41.37 ± 5.11^a^
Isobutyric acid (*μ*g/g)	56.33 ± 23.99^a^	15.12 ± 7.61^b^	6.06 ± 1.20^b^	6.40 ± 0.91^b^
Total SCFAs (*μ*g/g)	260.41 ± 56.49^a^	255.05 ± 52.26^a^	200.84 ± 25.71^a^	232.36 ± 39.91^a^

Con group, administration of saline solution; Fru-L group, administration of a low dosage of fructose (2.6 g/kg/day); Fru-M group, administration of a moderate dosage of fructose (5.3 g/kg/day); Fru-H group, administration of a high dosage of fructose (10.5 g/kg/day). Data are expressed as the mean ± standard deviation (*n* = 10). Values in the same row that do not share the same lowercase letter are significantly different (*P* < 0.05).

## Data Availability

The data used to support the findings of this study are available from the corresponding author upon request.
